# ERCP Following Laparoscopic Cholecystectomy: A Safe and Effective way to Manage CBD Stones and Complications

**DOI:** 10.1155/1995/58905

**Published:** 1995

**Authors:** Colleen M. Schmitt, John Baillie, Peter B. Cotton

**Affiliations:** 1 Division of Gastroenterology University of Tennessee Chattanooga Tennesse USA; 2 Division of Gaproenterocopy Duke University Medical Center Durham North Carolina 27710 USA; 3 Division of Gastroenterology Medical University of South Carolina Charleston South Carolina USA

## Abstract

The efficacy of ERCP in detecting and treating post-laparoscopic cholecystectomy problems was examined in a series of consecutive patients undergoing directed examination of the biliary tree over a two-year period. Three major diagnostic groups were identified: leaks and bile duct injuries (*n* = 9), retained common bile duct stones (*n* = 18), and post-cholecystectomy pain (*n* = 13). These diagnostic groups differed in degree of abnormal bilirubin (*p* = .004) and time between surgery and ERCP (*p* = .0005). Diagnosis of a post-operative complication was successful in 92% of attempted cases. Therapy was successful in 92% of attempted cases. Three patients developed mild pancreatitis as a result of ERCP. This series underscores the efficacy of a multi-disciplinary approach to problems which occur after laparoscopic cholecystectomy.


*HPB Surgery*, 1995, Vol. 8, pp. 187–192
Reprints available directly from the publisher
Photocopying permitted by license only

© 1995 Harwood Academic Publishers GmbH
Printed in Malaysia

### ERCP Following Laparoscopic Cholecystectomy: A Safe and Effective way to Manage CBD Stones and Complications

COLLEEN M. SCHMITT*, JOHN BAILLIE† and PETER B. COTTON§

* Division of Gastroenterology, University of Tennessee, Chattanooga, Tennesse; † Division of Gaproenterocopy, Duke University Medical Center, Durham, North Carolina; § Division of Gastroenterology, Medical University of South Carolina, Charleston, South Carolina

The efficacy of ERCP in detecting and treating post-laparoscopic cholecystectomy problems was examined in a series of consecutive patients undergoing directed examination of the biliary tree over a two-year period. Three major diagnostic groups were identified: leaks and bile duct injuries ($n = 9$), retained common bile duct stones ($n = 18$), and post-cholecystectomy pain ($n = 13$). These diagnostic groups differed in degree of abnormal bilirubin ($p = .004$) and time between surgery and ERCP ($p = .0005$). Diagnosis of a post-operative complication was successful in 92% of attempted cases. Therapy was successful in 92% of attempted cases. Three patients developed mild pancreatitis as a result of ERCP. This series underscores the efficacy of a multi- disciplinary approach to problems which occur after laparoscopic cholecystectomy.

KEY WORDS: Laparoscopic cholecystectomy complications endoscopic retrograde cholangiopancreatography bile duct bile duct stricture

### INTRODUCTION

Laparoscopic cholecystectomy (LC) is the new gold standard for surgical treatment of symptomatic gall bladder disease[1]. Despite the absence of controlled trials comparing this relatively new procedure with standard open cholecystectomy (OC), LC is widely accepted because of decreased hospital stay, swift return to normal activities, reduced post-operative pain, and better cosmetic results[2-4].

With the rapid endorsement of LC, the role of the biliary endoscopist in diagnosis and management of two major post-operative problems is increasing. The most important of these is bile duct injury. Regional experiences with LC have demonstrated an increased rate of bile duct injury compared with OC. This rate appears disproportionately high in the early experience of the surgeon[5-7]. The second dilemma is retained common bile duct stones which require removal. Patients with these post-operative problems may be referred to the biliary endoscopist for diagnosis or therapy.

*Address for correspondence:* Box 3515, Duke University Medical Center, Durham, NC 27710

We report our experience in diagnostic and therapeutic ERCP in 52 consecutive patients referred to our endoscopy unit at a tertiary medical center following LC.

### MATERIALS AND METHODS

Patients were identified by a retrospective review of the Duke University Medical Center (DUMC) Gastrointestinal Endoscopy Database. Data were collected by chart abstraction and telephone interview for a 2 year period between March 1990 through February 1992. Criteria for entry were: (1) previous LC or LC conversion to OC, and (2) referral specifically for examination of the biliary tree. Demographic data included age, gender, and referral area. Operative data included results of intra-operative cholangiogram (if performed), and whether conversion to OC was required. Pre-ERCP data included number of days from surgery until referral, type of symptoms (e.g., fever, abdominal pain, fistulous drainage), requirement for percutaneous drainage of a biloma, and elevation of liver chemistries (upper limit of normal: AST 45 u/l, ALT 45 u/l, AP 110 u/l, TB 1.2 mg/dl). ERCP data included type of therapeutic procedure(s) performed, number of procedures required to complete therapy, duration of stenting, and type and outcome of other invasive therapy required (percutaneous or surgical). Success of therapeutic ERCP was defined as (1) avoidance of surgical correction, and (2) resolution of the specific problem leading to the procedure (e.g., resolution of leak or stricture, removal of retained common bile duct (CBD) stones). Complications were defined according to Cotton *et al.*[8] Statistical tests were performed using nonparametric methods (Wilcoxon Rank Sum test and Kruskal-Wallis test for multiple groups) as data were not assumed to have normal distribution.

### RESULTS

During this 2 year period, 58 patients were referred to the DUMC biliary endoscopy service. Six patients were excluded for examination not directed at the bile duct ($n$ = 5) and routine follow-up of primary sclerosing cholangitis ($n$ = 1). Fifty-two patients were included for study.

There were 19 males and 33 females. Median age was 43.5 years (range, 16–93 years). LC had been performed outside DUMC in 36 cases (69.2%). For the purposes of this review, patients were categorized into three major diagnostic groups: Group 1 = leaks and bile duct injuries, Group 2 = retained CBD stones, and Group 3 = post-cholecystectomy pain (post–LC pain) (Table 1). Patients most commonly presented with abnormal liver chemistries and abdominal pain (Table 2). Liver chemistries were elevated in 81% of patients with median (range), AST 43 u/l (5-400), ALT 51 u/l (1-440), AP u/l 162 (52-678), TB 0.8 mg/dl (0.1-9.0) (Table 3). The median delay between surgery and ERCP was 21 days (range, 1-354) (Table 4).

**Table 1** Diagnosis at ERCP by major diagnostic group ($n$ = 50)[a]

| | | *N* | *(%)* |
|---|---|---|---|
| Group 1 | Injuries | 19 | (38) |
| | Leaks (12) | | |
| | Strictures (7) | | |
| Group 2 | Retained CBD stones | 18 | (36) |
| Group 3 | Post-LC pain | 13 | (26) |
| | SOD (2) | | |
| | Pancreatitis (1) | | |
| | Normal (10) | | |

[a] Two patients failed diagnostic ERCP

**Table 2** Clinical presentation ($n$ = 52)[a]

| | *N* | *(%)* |
|---|---|---|
| Abnormal liver chemistries | 42 | (81%) |
| Abdominal pain | 22 | (42%) |
| Abnormal cholangiogram | 11 | (21%) |
| Percutaneous drainage of fluid collection | 11 | (21%) |
| Known or suspected injury at the time of surgery | 5 | (10%) |

[a] Some patients presented with more than one clinical finding or indication for ERCP

**Table 3** Liver chemistries by diagnostic group, median (range)

| | *Group 1* *n = 18* | *Group 2* *n = 18* | *Group 3* *n = 14* | *Overall* *n = 52* |
|---|---|---|---|---|
| AST[a] | 36 (5-265) | 61 (12-400) | 37 (8-81) | 43 (5-400) |
| ALT[b] | 46 (1-440) | 75 (7-428) | 32 (11-112) | 51 (1-440) |
| AP[c] | 162 (52-678) | 172 (55-575) | 111 (55-391) | 162 (52-678) |
| TB[d] | 0.8 (0.3-6.0) | 2.2 (0.2-9.0) | 0.6 (0.1-1.9) | 0.8 (0.1-9.0) |

[a] $p$ = .09, Kruskal-Wallis

[b] $p$ = .07, Kruskal-Wallis

[c] not statistically significant, $p$ = .38, Kruskal-Wallis

[d] $p$ = .004, Kruskal-Wallis

**Table 4** Time from surgery to ERCP, days ($n$ = 52)

| | *Median* | *Range* |
|---|---|---|
| Group 1 | 19.0 | 3-337 |
| Group 2 | 7.5 | 1-354 |
| Group 3 | 118.0[a] | 7-227 |
| Overall | 21.0 | 1-354 |

[a] $P$ = .0005, Kruskal-Wallis

*Group 1.*

Nineteen patients were diagnosed with bile duct injury or bile duct leak by ERCP. The median time from surgery until ERCP was 19 days (range, 3-337) (Table 4). Abnormal liver chemistries were present in 15 (79%): median (range) AST 36 u/l (5-265), ALT 46 u/l (1-440), AP 162 u/l (52-678), TB 1.4 mg/dl (0.3-6.0). Cholestasis without jaundice was the most frequent finding with elevations of AP in 67%. Transaminases were mildly elevated (1.5X normal) in 40% of patients. Ten patients (52.6%) had an abdominal fluid collection (biloma) requiring CT-guided drainage. Five patients (26%) had attempted repair of the injured site or biliary-enteric bypass acutely and were referred for ERCP with suspected complication.

The cystic duct (CD) stump was the most common site of bile leak ($n$ = 9) (Table 5). A disrupted CBD ($n$ = 1), CBD

**Table 5** Injury site, N

| | |
|---|---|
| LEAK | |
| Cystic duct | 9 |
| Common bile duct | 2 |
| Right hepatic duct[a] | 2 |
| STRICTURE | |
| Common bile duct, complete | 2 |
| Common bile duct, partial | 2 |
| Common hepatic duct | 3 |

[a] 1 patient diagnosed and treated at ERCP; 1 patient failed diagnostic ERCP and was treated precutaneously leak ($n = 1$) and RHD leak ($n = 1$) were identified. Complete obstruction of the CBD was seen in 2 patients. Strictures were identified, in the CBD in 2 patients and CHD in 3 patients.

Endoscopic therapy was initiated in 12 patients (Table 6). Six cases of bile duct injury or leak were treated with endoscopic prosthesis (EP). Leaks from the CD stump ($n = 5$) and RHD ($n = 1$) were stented for a median duration of 37 days. Three patients with strictures of the CBD ($n = 2$) and CHD ($n = 1$) underwent a trial of EP for a median duration of 96 days. EP was successful in 5 of 6 leaks (83%) and 2 of 3 strictures (67%). Three of 3 leaks (100%) were successfully treated by ES alone. Two patients underwent endoscopic sphincterotomy (ES) for concomitant stone extraction. ES was not required for stent placement.

Surgical correction was performed as primary therapy in 6 patients: hepato-jejunostomy was performed for complete CBD obstruction ($n = 2$), CHD stricture ($n = 2$), and disrupted CBD ($n = 1$); 1 patient with a leak of the CD stump underwent surgical repair.

Diagnostic ERCP failed in one patient who presented with a biloma; a RHD leak was indentified by percutaneous cholangiogram and the patient underwent hepato-jejunostomy. Access for therapeutic ERCP failed in one patient who presented with a dislodged tube which had been placed in the CBD at surgery. At that time, complete transection of the CBD was recognized and immediate choledochoduodenostomy was performed. The patient was successfully treated with a percutaneously placed stent. EP failed in the patient with the RHD leak who underwent subsequent Longmire procedure. EP (total duration, 300 days) failed in one CBD stricture which occurred after primary repair of a transected duct. This patient underwent successful hepato-jejunostomy.

Overall, definitive therapy was provided by EP in 7 of 9 patients (78%) and ES in 3 of 3 patients (100%). Overall success for attempted therapy in this group was 83% (Table 6).

**Table 6** Summary of ERCP results ($n = 52$)

| | *Success* | *Failure* | *Complications* |
|---|---|---|---|
| Diagnostic | | | |
| Group 1 | 6 | 1 | 0 |
| Group 2 | 4 | 0 | 0 |
| Group 3 | 13 | 1 | 2 |
| Total | 23 (92%) | 2 (8%) | 2 [a] |
| Therapeutic [b] | | | |
| Group 1 | 10 | 2 | 0 |
| Leak (EP) | 5 | 1 | 0 |
| (ES) | 3 | 0 | 0 |
| Stricture (EP) | 2 | 1 | 0 |
| Group 2 | 14 | 0 | 1 |
| Group 3 | – | – | – |
| Total | 24 (92%) | 2 (8%) | 1 [a] |

[a] Mild pancreatitis

[b] Access for therapy failed in 1 patient

*Group 2.*

Eighteen patients were suspected to have retained bile duct stones causing abnormal liver chemistries, abdominal pain, or an abnormal cholangiogram. Abnormal liver chemistries were present in 12 (67%): median (range) AST 61 u/k (12-400), ALT 75 u/l (7-428), AP 172 u/l (55-575), and TB 2.2 mg/dl (0.2-9.0). Cholestasis with jaundice was most common, with elevations of alkaline phosphatase and bilirubin in >60% of patients. Nine patients had suspected bile duct stones on the basis of an abnormal cholangiogram; one of these was found to have a clear bile duct at the time of ERCP. Retained stones were confirmed in an additional 6 patients. Three patients were found to have a clear bile duct at the time of ERCP, however, the papilla appeared "ragged", raising the suspicion that a stone had recently passed. A total of 14 patients were treated for retained bile duct stones: 12 of 14 (86%) required ES;5/14 (36%) required more than one procedure to clear the bile duct. Overall success of therapy in this group of patients was 100%.

*Group 3.*

Fourteen patients were evaluated for asymptomatic elevations of liver chemistries (n=1) or post-LC pain (n=13). Abnormal liver chemistries were present in 4 (28.5%): median (range) AST 37 u/l (8-81), ALT 32 u/l (11-112), AP 111 u/l (55-391), TB 0.55 mg/dl (0.1-1.9). Diagnostic ERCP failed in 1 patient who could not tolerate the procedure. Two patients had evidence for papillary stenosis with delayed drainage of contrast from the bile duct; no therapy was undertaken in one while one patient underwent surgical sphincteroplasty. One patient had evidence of pancreatitis and normal bile ducts. The remaining 10 patients had normal bile ducts.

For this entire series, diagnostic procedures alone were attempted in 25 patients; therapeutic procedures were attempted in 27 cases. Diagnostic ERCP failed in 2 of 25 cases (8%). Access for therapy failed in 1 of 27 cases (3.7%). Three complications occurred in this series. Two patients evaluated for post-LC pain developed

**Table 7** Summary of Results

| | *Success by intent* | *Success for therapy* |
|---|---|---|
| Group I | 18/20 (90%) | 10/12 (83%) |
| Group II | 18/18 (100%) | 14/14 (100%) |
| Group III | 13/14 (92.8%) | – |
| Overall | 49/52 (94.2%) | 24/26 (92.3%) |

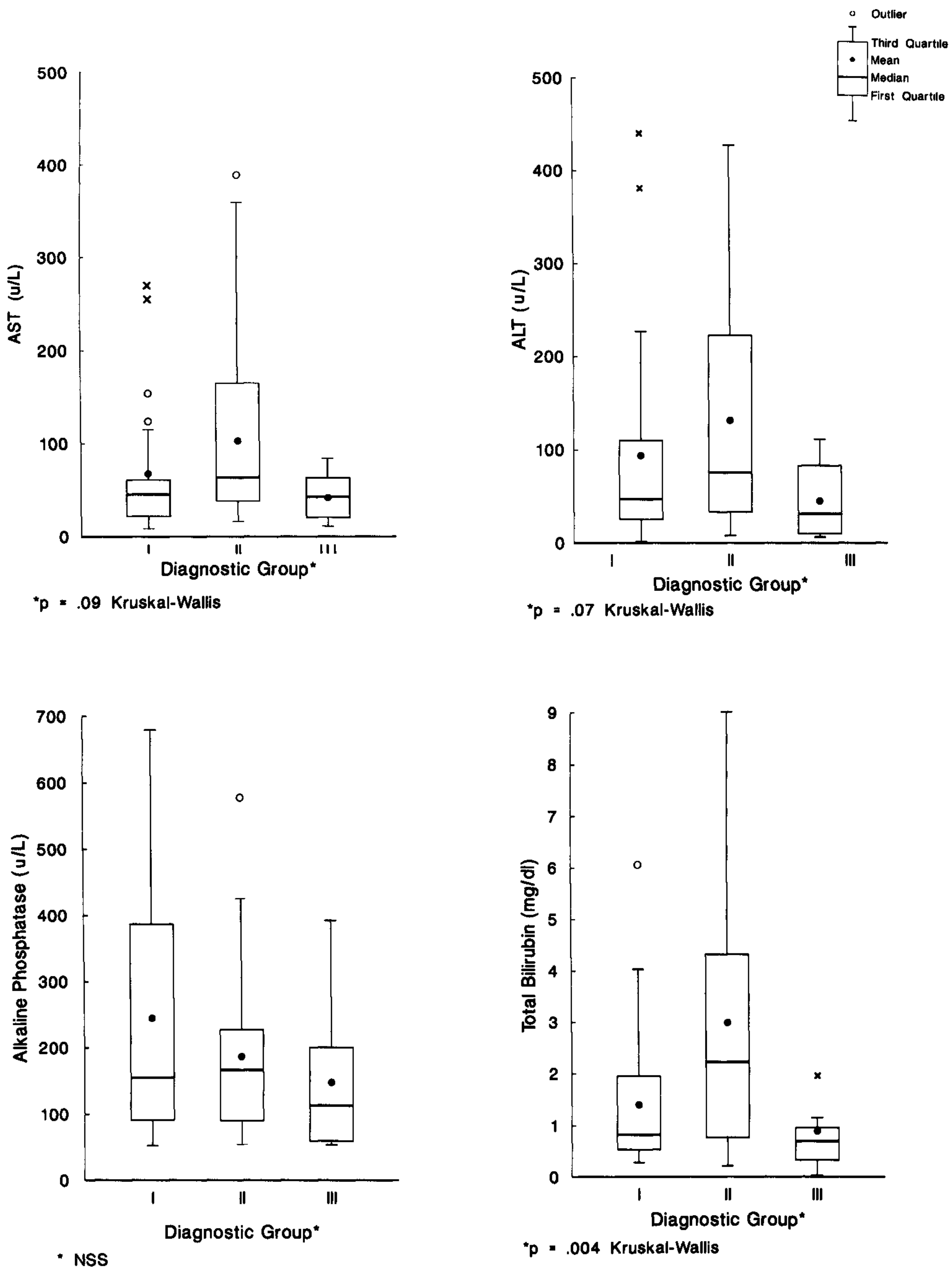

**Figure 1** Liver chemistries by diagostic group.

mild pancreatitis. One patient who underwent ES and stone extraction with interim stent placement developed mild pancreatitis (Table 7).

Differences in liver chemistry abnormalities were statistically significant between the 3 major diagnostic groups.(Table 3 and Figure 1) The difference in delay time between surgery and ERCP for the 3 major diagnostic groups was very highly statistically significant, $p = .0005$ (Figure 2).

### DISCUSSION

In the absence of prospective, randomized trials, the enthusiasm for LC has been tempered by careful scrutiny of associated complications. Appeals for circumspection in the use of the procedure and credentialing of trainees are now familiar[9-10]. Attention is turning to the long-term outcome and management of complications after LC. The therapeutic endoscopist can serve an important role in the

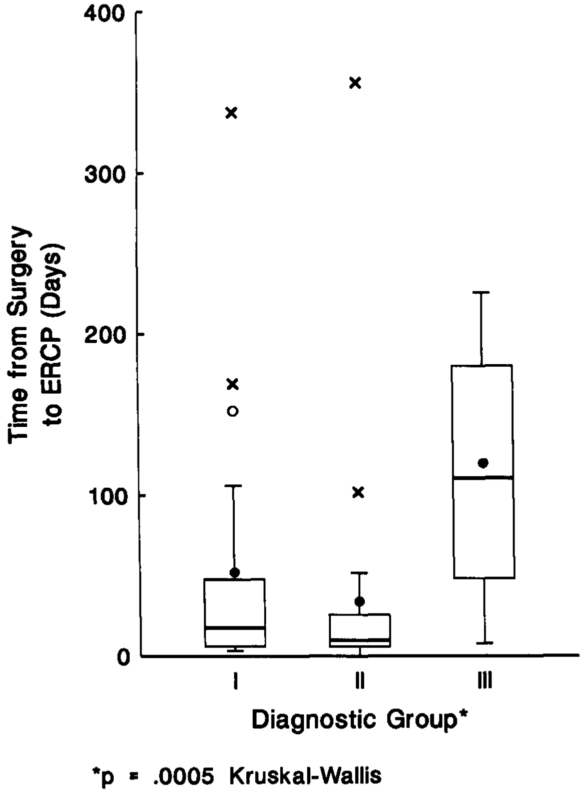

**Figure 2** Time from surgery to ERCP (days).

management of post-operative dilemmas as described in this case series.

Bile duct injury has emerged as the most serious complication of LC and appears related to early experience[2,5 6]. The classic pattern of injury occurs when the common bile duct is mistaken for the cystic duct and a portion of the misidentified duct is then removed. The major post-operative manifestations are bile leak and biliary obstruction[11 12].

The management strategy for injuries which are suspected post-operatively will depend on the expertise available. Adequate visualization of the leak site, percutaneous drainage of fluid collections, and relief of distal obstruction are rules for successful management[13]. Radiologic and endoscopic management have the advantage of avoiding further surgical morbidity.

Percutaneous transhepatic drainage provides definitive management for biliary fistulae in 50–80% of cases[14 15]. This method may also temporize by diverting bile flow away from the bile duct defect until the patient's clinical condition improves for surgical repair. However, in addition to the risk of bleeding due to transhepatic puncture, percutaneous drainage may be more difficult in this group of patients since the bile ducts are effectively decompressed. For these reasons, we reserve this approach for patients who have failed endoscopic access.

Success rates exceeding 75% for ES and EP have been demonstrated for management of post-operative biliary fistulae[13,16]. The endoscopic management of similar sequelae after LC appears to be increasing. Wootton reported successful treatment of 2 of 3 cystic duct leaks treated with EP[17]. Kozarek *et al.*, have reported the most complete series to date. Three CD leaks, 6 CBD leaks, and 2 CBD transections were identified at ERCP. Bile leaks sealed in the 9 patients treated endoscopically. The discrete referral base and consecutive nature of the patient sample allowed better characterization of the range of injuries and management[6]. In our case series, we have demonstrated successful treatment in 5 of 6 patients (83%) with EP and 3 of 3 patients (100%) with ES. The similar success rates for ES and EP suggest an analogous mechanism: relief of relative obstruction at the sphincter of Oddi.[13] The retrospective nature and small number of cases limit valid comparisons of the two approaches. ES has been recommended in this setting because of infection and blockage associated with stents[18]. We prefer EP over ES to avoid the increased risk of bleeding, perforation, and pancreatitis associated with ES[8]. The limited duration of stent placement (median, 37 days) obviates the need for routine stent exchange since these complications are unlikely to occur. We do not routinely perform ES for placement of the stent.

High success rates for endoscopic management of post-operative biliary strictures has been similarly demonstrated. Good to excellent results were shown by Davids, *et al.* in 83% of patients treated with EP for one year, however, restricturing occurred in 17%[19]. Accounts of this modality for managing post-LC strictures are limited to two reports. Weber treated a stricture related to a CD clip with EP[20]. Wootton's series included 2 CBD strictures; 1 of these was successfully treated with EP[17]. We attempted therapy in 3 patients with strictures after LC and achieved resolution in 2. Only long-term follow-up will define the success of this therapy. The reasons for limited endoscopic experience in this clinical setting are unclear. We have not attempted to describe our referral base, therefore the denominator and rate for this particular injury is undefined in our series. The referral pattern for complications of this relatively new surgery may be confined to the surgical community[12]. Complete or complicated obstruction will require expert surgical correction. However, our results suggest that a trial of endoscopic therapy is warranted in the appropriate clinical setting, as for any benign bile duct stricture.

Are retained bile duct stones "complications"? This is a matter of semantics. The endoscopic removal of retained stones is conventional; our success rate after LC is high (100%). Currently, attention is appropriately focused on pre-operative identification and management of patients with suspected choledocholithiasis[21-23].

Davidoff *et al.*, emphasized that prolonged or severe abdominal pain post-LC should heighten suspicion for a complication, since one of the benefits of LC is relative lack of post-operative discomfort[11]. We observed a distinct clinical presentation for the 3 major diagnostic groups based on timing of ERCP and degree of abnormal liver chemistries.[a] Patients with findings at ERCP of serious consequence (bile leaks, strictures and retained stones) underwent study within 1–2 weeks of surgery and liver chemistries were more strikingly abnormal. In contrast, patients in the group with post-LC pain had relatively unremarkable elevations in liver chemistries and presented 3.9 months after surgery. A low threshold for study is reflected in earlier referral for patients with objective evidence of significant disease (e.g., presence of biloma, abnormal cholangiogram, abnormal liver chemistries). Our review was retrospective and the sample was drawn from patients referred to a tertiary center for biliary endoscopy. We conclude that a low-threshold for diagnostic evaluation of potential post-operative complications should be maintained, especially in patients with abdominal pain or abnormal liver chemistries. This case series demonstrates that ERCP is ideally suited as an adjunct to laparoscopic surgery to address both diagnosis and therapy of complications.

Outcomes research defining the nature and long-term significance of post-LC complications is required. The prospect is both exciting and formidable. This series demonstrates an excellent example of multidisciplinary collaboration.

[a] This suspicion was confirmed by exploratory data analysis. Statistical tests were performed only on the measures discussed, therefore limiting the risk for a type I error from multiple comparisons.

### REFERENCES

1. Salky, B. (1992) Laparoscopic cholecystectomy, in Postgraduate Course, The American Society for Gastrointestinal Endoscopy.
2. Dubois, F., Icard, P., Berthelot, G., Levard, H. (1990) Coelioscopic cholecystectomy: preliminary report of 36 cases. *Annals of Surgery*, **211**, 60–62.
3. Vitale, G. C., Collet, D., Larson, G. M., Cheadle W. G., Miller F. B., Perissat, J. (1991) Interruption of professional and home activity after laparoscopic cholecystectomy among French and American patients. *American Journal of Surgery*, **161**, 396–398.
4. Grace, P. A., Quereshi, A., Coleman, J., Keane, R., McEntee, G., Broe, P., Osborne, H., Bouchier-Hayes, D. (1991) Reduced postoperative hospitalization after laparoscopic cholecystectomy. *British Journal of Surgery*, **78**, 160–162.
5. The Southern Surgeons Club. (1991) A prospective analysis of 1,518 laparoscopic cholecystectomies performed by Southern U.S. Surgeons. *The New England Journal of Medicine*, **213**, 3–12.
6. Kozarek, R., Gannan, R., Wagonfeld, J., Ball, T. (1991) Bile leak after laparoscopic cholecystectomy: Diagnostic and therapeutic application of endoscopic retrograde cholangiopancreatography. *Archives of Internal Medicine*, **152**, 1040–1043.
7. Peters, J. H., Ellison, E. C., Innes, J. T., Liss, J. L., Nichols, K. E., Lomano, J. M., Roby, S. R., Front, M.E., Carey, L. C. (1991) Safety and efficacy of laparoscopic cholecystectomy: a prospective analysis of 100 initial patients. *Annals of Surgery*, **213**, 3–12.
8. Cotton, P. B., Lehman, G., Vennes, J., Geenen, J. E., Russell, R. C. G., Meyers, W. C., Liguory, C., Nickl, N. (1991) Endoscopic sphincterotomy complications and their management: an attempt at consensus. *Gastrointestinal Endoscopy*, **37**, 383–393.
9. Cuschieri, A., Berci, G., McSherry, C.K. (1990) Laparoscopic cholecystectomy. *American Journal of Surgery*, **159**, 273.
10. Salky, B. A., Bauer, J. J., Kreel, I., Gelernt, I. M., Gorfine, S. R. (1991) Laparoscopic cholecystectomy: an initial report. *Gastrointestinal Endoscopy*, **37**, 1–4.
11. Davidoff, A.M., Pappas, T.N., Murray, E.A., Hilleren, D.J., Johnson, R.J., Baker, M.E., Newman, G.E., Cotton, P.B., Meyers, W.C. (1992) Mechanisms of major biliary injury during laparoscopic cholecystectomy. *Annals of Surgery*, **215**, 196–202.
12. Moossa, A. R., Easter, D. W., Van Sonnenberg, E., Casola, G., D'Agostino, H. (1992) Laparoscopic injuries to the bile duct: a cause for concern. *Annals of Surgery*, **215**, 203–208.
13. Ponchon, T., Gallez, J. F., Valette, P. J., Chavaillon, A., Bory, R. (1989) Endoscopic treatment of biliary tract fistulas. *Gastrointestinal Endoscopy*, **35**, 490–498.
14. Kaufman, S. L., Kadir, S., Mitchell, S. E., Chang, R., Kinnison, M. L., Cameron, J. L., White, R. I., Jr. (1985) Percutaneous transhepatic biliary drainage for bile leaks and fistulas. *American Journal of Roentgenology*, **144**, 1055–1058.
15. Papanicolaou, N., Mueller, P. R., Ferrucci, J. T., Jr., Dawson, S. L., Johnson, R.D., Simeone, J. F., Butch, R. J., Wittenberg, J. (1984) Abscess-fistula association: Radiologic recognition and percutaneous management. *American Journal of Roentgenology*, **143**, 811–815.
16. Liguory, C., Vitale, G. C., Lefebre, J. F., Bonnel, D., Cornud, F. (1991) Endoscopic treatment of postoperative biliary fistulae. *Surgery*, **110**, 779–783.
17. Wootton, F. T., Hoffman, B. J., Marsh, W. H., Cunningham, J. T. (1992) Biliary complications following laparoscopic cholecystectomy. *Gastrointestinal Endoscopy*, **38**, 183–185.
18. Ralph-Edwards, T., Himal, H.S. (1992) Bile leak after laparoscopic cholecystectomy. *Surgical Endoscopy*, **6**, 33–35.
19. Davids, P. H. P., Rauws, E. A. J., Coene, P. P. L. O., Tytgat, G. N. J., Huibregtse, K. (1991) Endoscopic stenting for postoperative biliary strictures. *Gastrointestinal Endoscopy*, **38**, 12–18.
20. Weber, J., Adamek, H. E., Riemann, J. F. (1992) Endoscopic stent placement and clip removal for common bile duct stricture after laparoscopic cholecystectomy. *Gastrointestinal Endoscopy*, **38**, 181–182.
21. Voyles, C. R., Petro, A, B., Meena, A. L., Haick, A. J., Koury, A. M. (1991) A practical approach to laparoscopic cholecystectomy. *American Journal of Surgery*, **161**, 365–370.
22. Joyce, W. P., Keane, R., Burke, G. J., Daly, M., Drumm, J., Egan, T. J., Delaney, P. V. (1991) Identification of bile duct stones in patient undergoing laparoscopic cholecystectomy. *British Journal of Surgery*, **78**, 1174–1176.
23. Flowers, J. L., Zucker, K. A., Graham, S. M., Scovill, W. A., Imbembo, A. L., Bailey R. W. (1992) Laparoscopic cholangiography: results and indications. *Annals of Surgery*, **215**, 209–216.

